# Preparation and Performance Evaluation of Environmentally Friendly Foam Hydrogel Based on Polyvinyl Alcohol/Organic Titanium Crosslinking Agent

**DOI:** 10.3390/gels11030181

**Published:** 2025-03-06

**Authors:** Ru Ma, Gaoshen Su, Ya Nie, Huan Yang, Xiaorong Yu

**Affiliations:** 1 College of Chemistry and Environmental Engineering, Yangtze University, Jingzhou 434023, China; 2022710241@yangtzeu.edu.cn (R.M.); yanghuan@yangtzeu.edu.cn (H.Y.); yxr_cjdx@163.com (X.Y.); 2 China Oilfield Services Limited, Shenzhen 518000, China; nieya@cnooc.com.cn

**Keywords:** in situ foaming, environmentally friendly gel, adjustable strength, polyvinyl alcohol, foam gel

## Abstract

Foam and hydrogel profile control are commonly utilized water-blocking and profile modification techniques in oil fields. This study integrates a foam system with a gel system, employing an organic titanium crosslinking agent to crosslink polyvinyl alcohol, thereby forming a gel system. Concurrently, a gas-evolving agent is incorporated into the system to induce in situ foaming, thereby creating an environmentally benign foam gel system. The fundamental constituents of this system comprise 2 wt% to 5 wt% polyvinyl alcohol, 2 wt% to 4 wt% crosslinker, and 0.3 wt% to 0.9 wt% gas-generating agent. By varying the amounts of each component, the strength grade, gelation time, and foaming volume of the foam gel can be effectively adjusted. The results of the temperature resistance performance evaluation indicate that within the temperature range of 80 °C to 130 °C, the gelation performance of the foam gel is stable and good. At 90 °C, the foam gel can remain stable for 340 days with minimal strength variation. The plugging experiments indicate that the formulated foam gel system exhibits superior injectability and can effectively seal the sand-filled tube model, achieving a blocking efficiency of up to 96.36%.

## 1. Introduction

In recent years, the majority of China’s oil reservoirs have entered the secondary or tertiary oil recovery phases, with profile adjustment becoming a crucial measure to ensure the efficiency of oil displacement [1,2]. Oil fields commonly employ the method of injecting profile modification and blocking agents to seal high-permeability layers or zones. This approach adjusts reservoir heterogeneity, expands the swept volume, and thereby achieves the goal of increasing production [3,4,5]. In recent years, gel and foam profile modification and blocking agents have gained extensive on-site application owing to their pronounced modification and blocking effects [6,7].

Foam profile modification and blocking agents deform when passing through formation pore throats smaller than their diameter, thereby creating resistance to fluid flow. This mechanism effectively seals preferential flow channels for water, consequently expanding the sweep volume of the injected water. However, the foam profile modification system exhibits relatively low plugging strength and a limited duration of action. Consequently, this system’s capabilities for water control and efficiency enhancement are insufficient [8,9]. Gel-type profile control agents form a three-dimensional network structure through polymer crosslinking, characterized by high strength and robust stability. However, the volume of reservoir affected by these agents is relatively limited [10,11,12,13]. Foam gel is a three-phase colloidal system that combines foam and gel systems. The dispersion of gas phase in the liquid phase endows the foam gel with a highly porous structure, which typically has a high porosity. The gel matrix (solid phase) forms a three-dimensional network structure through crosslinking, which acts as the skeleton of the foam, fixing the bubble structure of the foam and enhancing its stability and mechanical strength. The physicochemical properties of the foam gel system are tunable. The porosity, pore size distribution, mechanical properties, and functional characteristics of the foam gel material can be adjusted by varying the concentration of the gel matrix, the concentration of the crosslinking agent, and the foaming method to meet the requirements of different applications [14,15].

The fabrication of foam gel blocking agents typically involves a gel base liquid augmented with surfactants that act as foaming agents. Foaming is induced either through mechanical high-speed agitation or by the injection of gas under similar stirring conditions. Implementing this foam gel preparation method on-site requires the deployment of specific mechanical equipment, which incurs additional construction costs and does not ensure the consistency of foam quality [16,17,18]. In response to this issue, researchers including Wang J [19], Hu X [20], and Telin [21] have conducted studies on self-gas-evolving foam gel blocking technology. They integrated self-gas-evolving agents with gel matrices to develop a composite system that generates foam with enhanced uniformity, stability, and longevity, eliminating the need for additional gas injection.

As reported in the literature [17], self-gas-evolving foam gels are primarily fabricated using polyacrylamide or inorganic binders as the matrix polymer. The crosslinkers typically employed in this system include glutaraldehyde, organic chromium crosslinkers, and borax, among others. Nevertheless, the latent toxicity of polyacrylamide and similar substances poses a risk to the ecological environment. Substituting polyacrylamide with polyvinyl alcohol as the gel matrix polymer can mitigate environmental damage. Organic titanium crosslinking agents are recognized for their low toxicity and are extensively utilized in biomedical applications, posing minimal environmental impact. The utilization of organic titanium crosslinking agents to crosslink polyvinyl alcohol in forming gel plugging agents is more aligned with environmental protection development concepts.

This study integrates a foam system with a gel system, employing polyvinyl alcohol as the matrix polymer and organic titanium as the crosslinking agent. Additionally, a gas-generating agent is incorporated to induce in situ foaming, resulting in the preparation of a series of environmentally friendly polyvinyl alcohol foam gels. The stability and plugging ability of these gels were evaluated.

## 2. Results and Discussion

### 2.1. Preparation of Polyvinyl Alcohol Foam Gels

#### 2.1.1. Optimization of Polyvinyl Alcohol Types

In this study, three types of polyvinyl alcohols (PVAs) with a polymerization degree of about 1700 and hydrolysis degrees of 88%, 97%, and 99% (PVA 1788, PVA 1797, and PVA 1799) were evaluated. Solutions of 5 wt% PVA 1788, PVA 1797, and PVA 1799 were prepared, and their viscosities were measured. For detailed results, see Table 1.

As illustrated in Table 1, PVA 1799 exhibits the highest viscosity under the same concentration conditions. The higher viscosity indicates that the intermolecular interactions within the polyvinyl alcohol solution are more significant, including hydrogen bonding and chain entanglement, which facilitate the formation of a more robust gel network when exposed to the crosslinking agent.

#### 2.1.2. Effect of Polyvinyl Alcohol and Crosslinker Concentration

**Figure 1:**
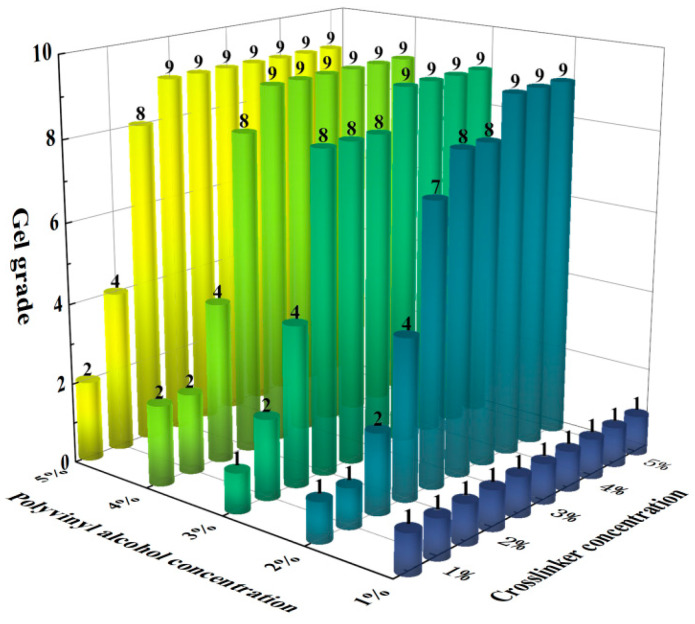
Effect of the synergistic effect of polyvinyl alcohol and crosslinker concentration on the gelatinization grade of foam gel.

**Figure 2:**
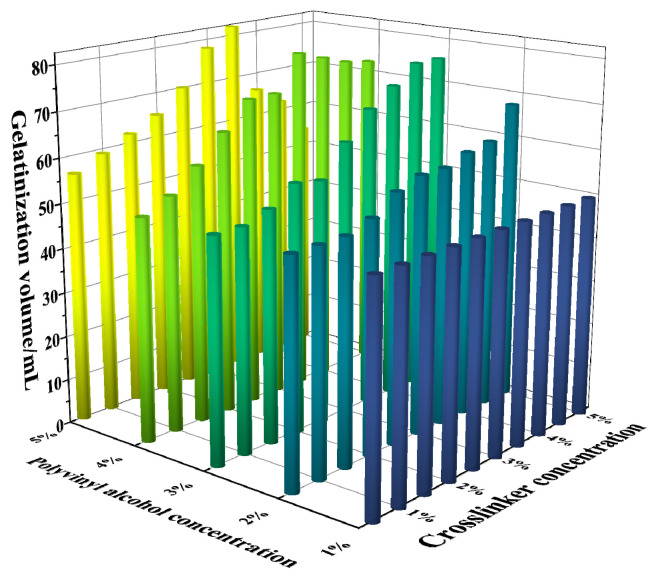
Effect of the synergistic effect of polyvinyl alcohol and crosslinker concentration on the gelatinization volume of foam gel.

This study prepared 50 mL of polyvinyl alcohol (PVA) foam gel base solutions with varying concentrations, with PVA concentration gradients ranging from 1 wt% to 5 wt%. The amount of crosslinking agent added varied from 0.5 wt% to 5 wt%, while the gas-generating agent was fixed at 0.5 wt%. Gelation experiments were conducted at 80 °C to investigate the synergistic effect of PVA and crosslinking agent concentrations on the performance of foam gels. Gel strength grades were categorized from A to J, where grade A corresponds to the lowest gel strength (grade 1), and grade J corresponds to the highest gel strength (grade 10). The experimental results are presented in Figure 1 and Figure 2.

**Figure 3:**
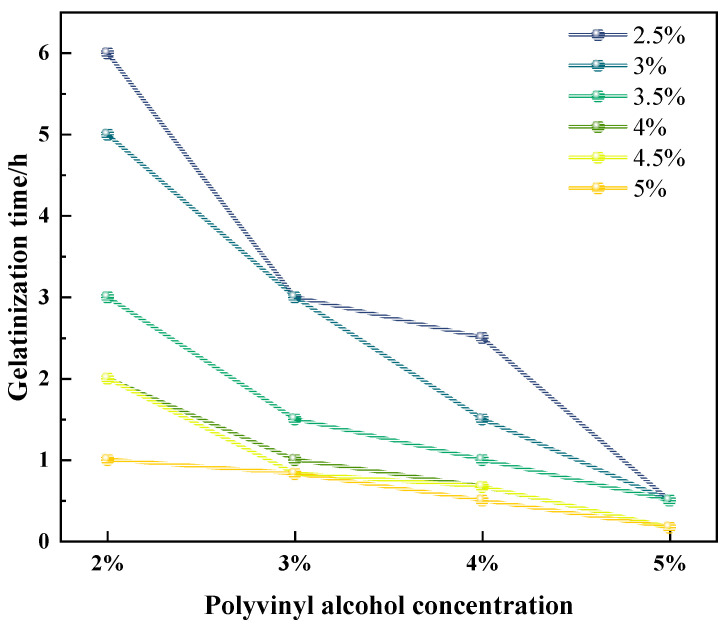
Effect of the synergistic effect of polyvinyl alcohol and crosslinker concentration on the gelatinization time of foam gel.

Figure 1 and Figure 2 illustrate the gelation grades and volumes of foam gels prepared at different concentrations of polyvinyl alcohol (PVA) and crosslinking agents. The experimental results indicated that gels produced at a polyvinyl alcohol concentration of 1% or a crosslinker concentration of 0.5–2% sometimes failed to meet the strength requirements of Class F. Based on these findings, we selected conditions with PVA concentrations ranging from 2 wt% to 5 wt% and crosslinking agent concentrations from 2.5 wt% to 5 wt% and further plotted the relationship between PVA concentration and gelation time, as shown in Figure 3.

**Figure 4:**
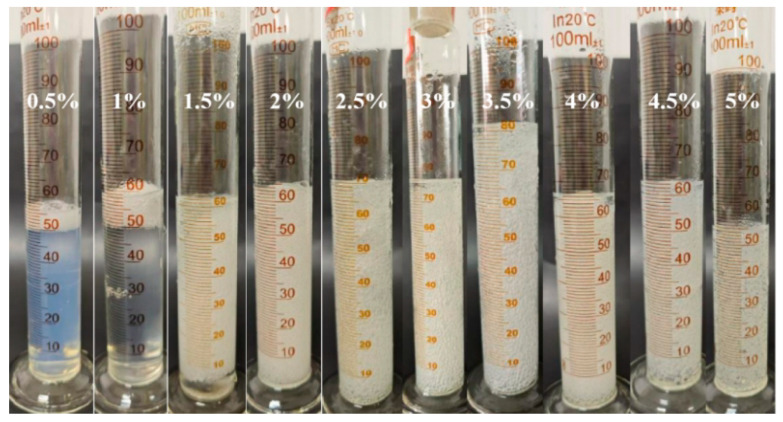
Experimental phenomena of gelatinization of 5 wt% polyvinyl alcohol concentration under different crosslinker concentrations.

As illustrated in Figure 1, Figure 2, Figure 3 and Figure 4, the gel strength, gelation time, and gel volume of foam gels can be adjusted within a certain range under different concentrations of polyvinyl alcohol (PVA) and crosslinking agents. When the crosslinking agent concentration is fixed, an increase in PVA concentration results in a decrease in gelation time, an increase in gel strength, and an initial increase followed by a decrease in gel volume. Similarly, when the PVA concentration is fixed, an increase in crosslinking agent concentration exhibits a similar trend. This trend is attributed to the formation of coordination bonds between titanium ions and ligands in the polyvinyl alcohol–organic titanium crosslinking agent system, which subsequently react with the hydroxyl groups in PVA molecules to form a gel network. As the PVA concentration increases, the number of hydroxyl groups available for complexation reactions also increases, leading to enhanced gel strength. Concurrently, the increased PVA concentration facilitates more interactions between hydroxyl groups and coordination bonds, thereby accelerating the gelation rate and reducing the gelation time. As the concentrations of PVA and crosslinking agent increase, the gel volume decreases. This is primarily because the rapid gelation and increased gel strength limit the migration and dispersion of gases within the gel system.

#### 2.1.3. Effect of Gas-Generating Agent Concentration

**Figure 5:**
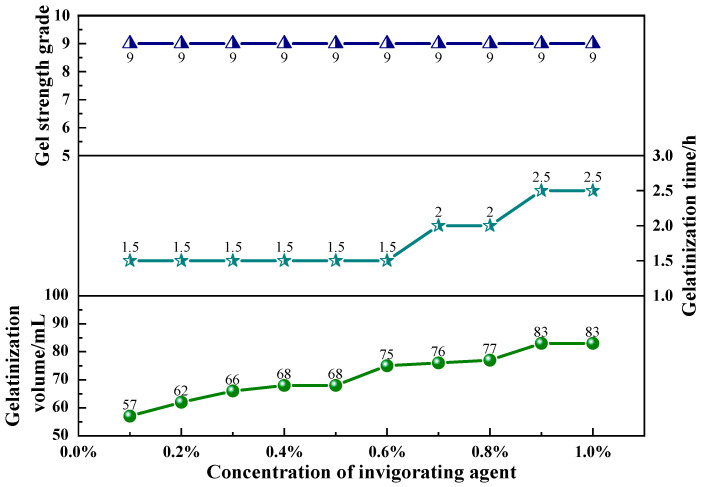
The effect of gas-generating agent concentration on the gel strength, gelation time, and gel volume of foam gels.

**Figure 6:**
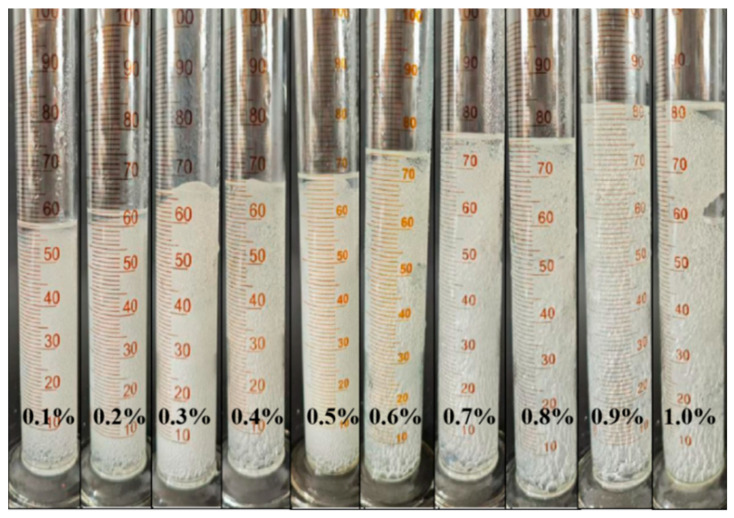
Experimental phenomena of foam gel formation under different gas-generating agent concentrations.

The present study formulated 50 mL of a polyvinyl alcohol foam gel base solution, consisting of 4 wt% polyvinyl alcohol (PVA) and 3 wt% titanium crosslinking agent (TiJ). The concentration of the gas-generating agent (V-50) was adjusted from 0.1 wt% to 1 wt%, with increments of 0.1 wt%, to investigate the effect of the gas-generating agent concentration on the performance of the foam gel. The experimental results are presented in Figure 5 and Figure 6.

As illustrated in Figure 5 and Figure 6, the increase in the concentration of the gas-evolving agent did not lead to changes in gel strength; the gelation time initially increased and then stabilized; the gel volume expanded with the increasing concentration of the gas-evolving agent, reaching a maximum at 0.9 wt%. At a concentration of 1.0 wt%, the excessive concentration of the gas-generating agent resulted in an overproduction of gas, which increased the disruptive effect on the gel, leading to gel rupture. The experimental results indicated that by adjusting the amount of the gas-generating agent added, the gel volume of the polyvinyl alcohol foam gel could be controlled.

### 2.2. Characterization of Polyvinyl Alcohol Foam Gels

#### 2.2.1. FTIR

**Figure 7:**
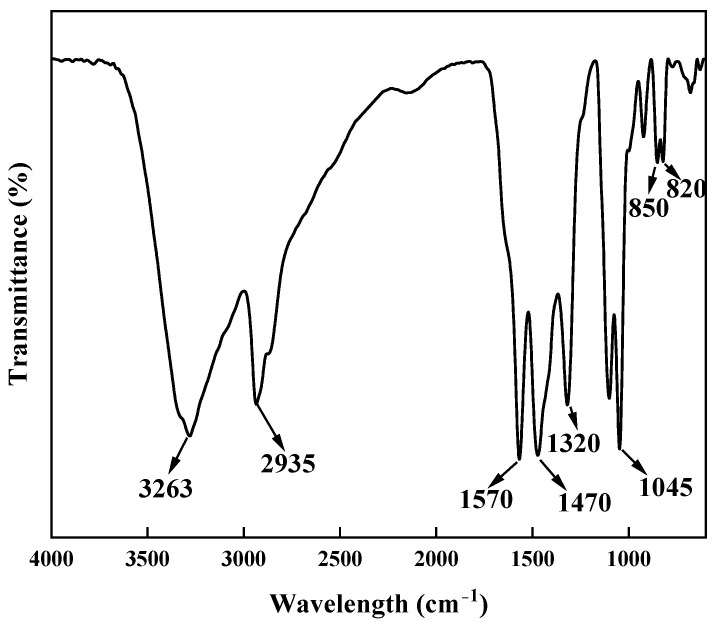
FTIR analysis of PVA foam gel.

In this study, the gel samples containing 4 wt% polyvinyl alcohol (PVA), 3 wt% titanium crosslinker (TiJ), and 0.9 wt% gas-generating agent (V-50) were subjected to freeze-drying treatment. The main functional groups were tested and analyzed using Fourier transform infrared spectroscopy (FTIR) with the potassium bromide (KBr) pellet method, and the results are presented in Figure 7.

As shown in Figure 7, the peaks between 3100 and 3400 cm^−1^ are attributed to the stretching vibrations of intermolecular and intramolecular hydrogen-bonded O-H groups. The peaks between 2800 and 3000 cm^−1^ are due to the stretching vibrations of alkyl C-H bonds. The peaks between 1550 and 1600 cm^−1^ are due to the stretching vibrations of C=O bonds in the organic titanium crosslinker. The peaks between 1300 and 1500 cm^−1^ are related to the bending vibrations of C-H bonds in the organic titanium crosslinker. The peaks between 1000 and 1100 cm^−1^ correspond to the stretching vibrations of C-O bonds in polyvinyl alcohol, and the peaks between 800 and 900 cm^−1^ are due to the Ti-O-C bonds formed between the organic titanium crosslinker and polyvinyl alcohol during crosslinking.

#### 2.2.2. SEM

**Figure 8:**
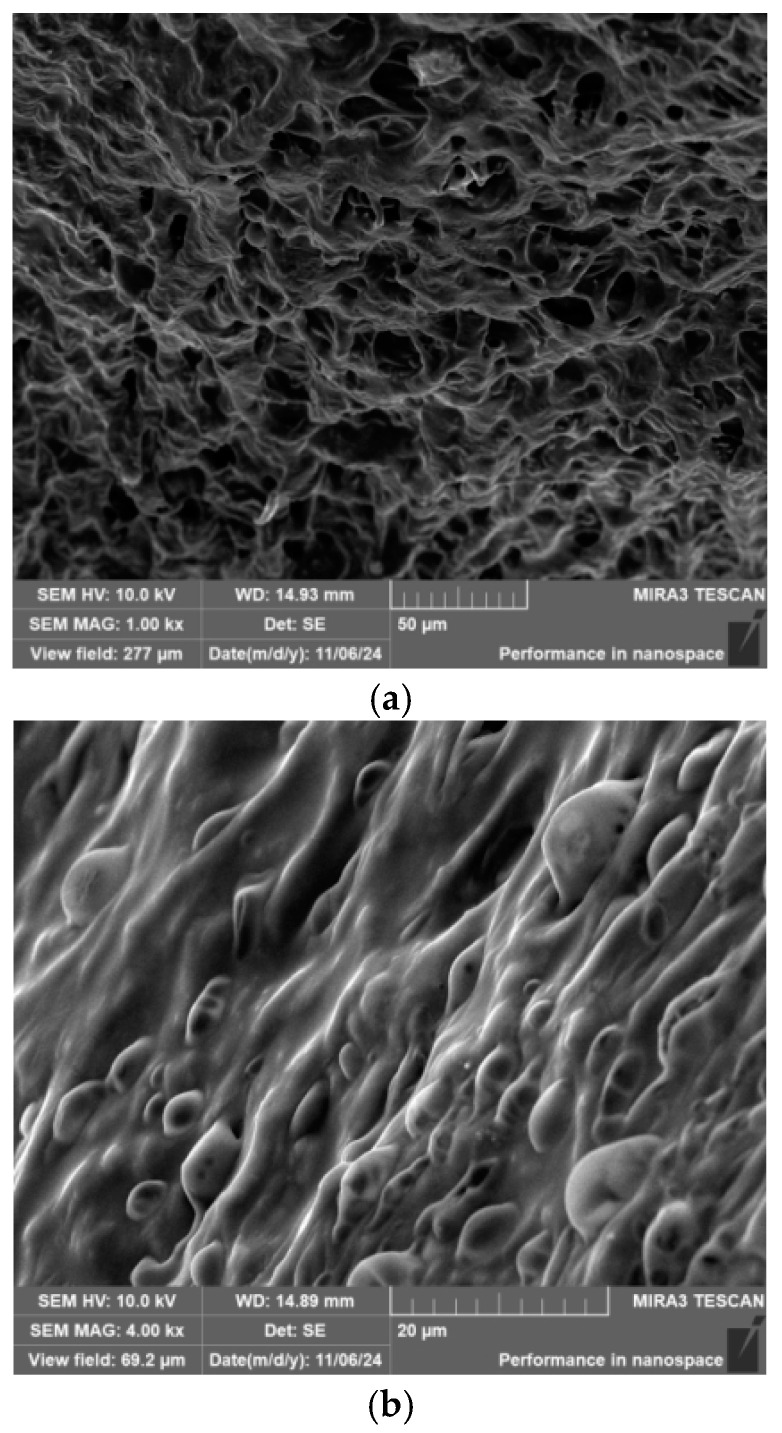
SEM: foam gel microstructure. (**a**) Internal structure of the gel; (**b**) Gel surface structure.

In this study, gel samples containing 4 wt% polyvinyl alcohol (PVA), 3 wt% titanium crosslinking agent (TiJ), and 0.9 wt% gas-generating agent (V-50) were subjected to freeze-drying treatment. Prior to observation under the field emission scanning electron microscope (SEM), the samples were gold-sprayed to enhance conductivity. The microstructure of the polyvinyl alcohol foam gel is presented in Figure 8.

As illustrated in Figure 8a, after freeze-drying, the bubble structure of the foam gel ruptures, revealing numerous pore structures. The skeleton between the pores is formed by the three-dimensional network structure of the polyvinyl alcohol gel post-gelation. This robust skeletal structure effectively reduces the rate at which bubbles burst within the foam gel, significantly enhancing its stability. During transport through porous media, the viscosity of the foam gel system gradually increases, forming a viscoelastic shell. This shell improves the mobility of water on the foam film and reduces the rate of liquid release from the foam, thereby enhancing foam stability [22]. After gelation, the three-dimensional network structure formed by the crosslinking reaction encapsulates the bubbles, creating a high-strength foam capable of effectively sealing high-permeability reservoirs. Moreover, when bubbles rupture, the released gas increases the formation energy, and the residual gel maintains a high viscosity, ensuring a high sealing capability.

In addition, as illustrated in Figure 8b, the self-generated bubbles produced during the crosslinking process further enhance the pore structure of the gel. After freeze-drying, some bubbles on the surface of the foam gel remain intact, with their size essentially maintained at 2 to 8 μm.

#### 2.2.3. Microstructure of the Gel

**Figure 9:**
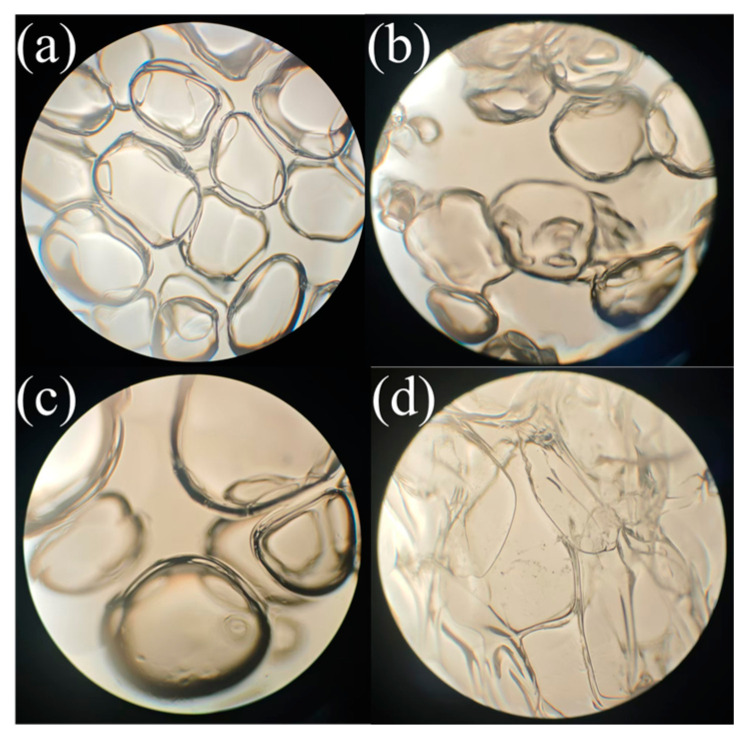
Morphological characteristics of foam gels at 4× magnification. (**a**) Initial state of polyvinyl alcohol foam gel; (**b**) State of polyvinyl alcohol foam gel after 100 days at 90 °C; (**c**) State of polyvinyl alcohol foam gel after 200 days at 90 °C; (**d**) State of polyvinyl alcohol foam gel after 362 days at 90 °C.

Under a polarizing microscope, the initial state of the foam gel (containing 4 wt% polyvinyl alcohol, 3 wt% titanium crosslinking agent, and 0.7 wt% gas-generating agent) immediately after gelation and the surface morphology after 362 days of gelation were observed under a 4× magnification. The observation results are presented in Figure 9.

As shown in Figure 9, water (liquid phase) acts as the continuous phase, dissolving PVA and filling the gel matrix. The PVA molecular chains (solid phase) form a gel network through chemical crosslinking. The N_2_ (gas phase) generated from the decomposition of the gas-generating agent V-50 under high-temperature conditions is dispersed in the gel. As illustrated in Figure 9a, in the initial gelation state, the bubbles within the polyvinyl alcohol foam gel display an irregular distribution pattern, and the bubble sizes are uniform and consistent, surrounded by a thicker liquid film. These bubble distribution characteristics facilitate the formation of a porous network structure within the gel, thereby enhancing the gel’s water absorption capability and structural stability. As shown in Figure 9, a comparative analysis of the foam gel’s morphology in its initial state and after 362 days of aging indicates that the bubble size of the polyvinyl alcohol foam gel increased, the number of bubbles decreased, and the thickness of the liquid film reduced, yet it did not burst completely. This suggests that the gel’s presence significantly enhanced the strength and toughness of the liquid film, forming a protective layer which hindered the rupture process. This protective effect enables the liquid film to maintain a certain level of stability even when it thins.

#### 2.2.4. TG

**Figure 10:**
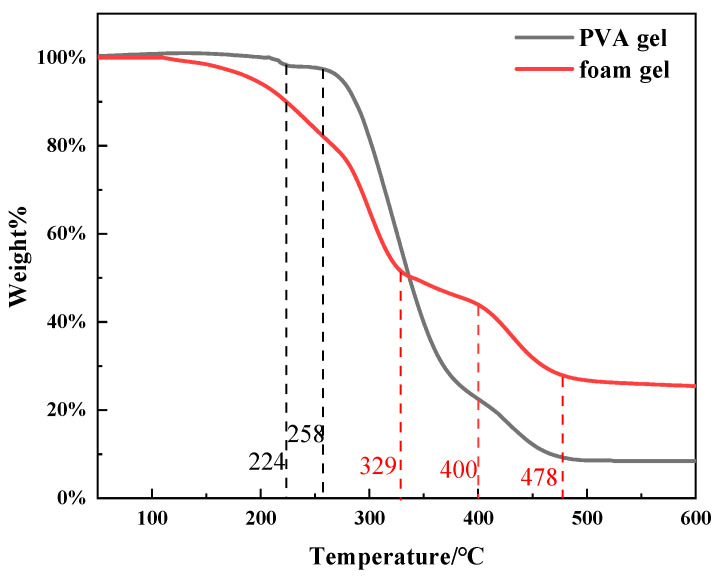
Weight loss rate–temperature variation of polyvinyl alcohol gel/foam gel.

This study utilized a thermal stability analyzer to assess the thermal stability of polyvinyl alcohol (PVA) and its foam gel system and generated the corresponding temperature–thermogravimetric (TG) curves, as presented in Figure 10. The experimental temperature range was established from 30 to 600 °C, and the thermal weight loss rate of the gel system with increasing temperature was characterized by monitoring the mass change.

As illustrated in Figure 10, PVA1799 experienced a minor mass loss prior to 224 °C, which is attributed to the residual water in the sample, accounting for about 1.5%. A significant mass loss was observed in the temperature range of 258 to 478 °C, which was due to the depolymerization and thermal degradation of the PVA molecular chain. Beyond 478 °C, the mass of the sample stabilized, primarily consisting of carbides and other inorganic residues, which constituted approximately 8.5%.

Within the temperature interval from 100 to 258 °C, the primary cause of mass loss in the foam gel was the evaporation of water. The moisture potentially contained within the bubbles of the foam structure led to a more pronounced water evaporation process in the foam gel compared to pure PVA, thereby resulting in a greater mass loss. In the temperature range of 258 to 329 °C, the mass loss rate of the PVA organic titanium crosslinked gel was slower compared to the pure PVA sample, indicating that the introduction of crosslinking points inhibited the thermal degradation process of the PVA molecular chain, thereby slowing down its depolymerization rate. In the temperature range of 329 to 400 °C, a new stage of mass loss was observed, which is attributed to the further decomposition of titanium-oxides formed in the crosslinking reaction at high temperatures, leading to the release of volatile substances. Some bubbles may have undergone thermal expansion or rupture at this stage, further accelerating the decomposition process of the gel. In the temperature range of 400 to 478 °C, the mass loss may have been due to the further decomposition of the organic components of PVA and the crosslinking agent, with the final residual components of the foam gel being approximately 26%. Compared to pure PVA, the residual amount of the foam gel increased, indicating that the introduction of crosslinking and titanium sources effectively increased the content of the charred residues of the gel. This suggests that the bubbles and gel network in the foam gel structure to some extent inhibit the complete degradation of PVA, indicating that the foam gel can still maintain a certain structural stability at high temperatures, which helps to maintain its overall performance [23].

### 2.3. Performance Testing of Polyvinyl Alcohol Foam Gels

#### 2.3.1. Effect of Salinity

**Figure 11:**
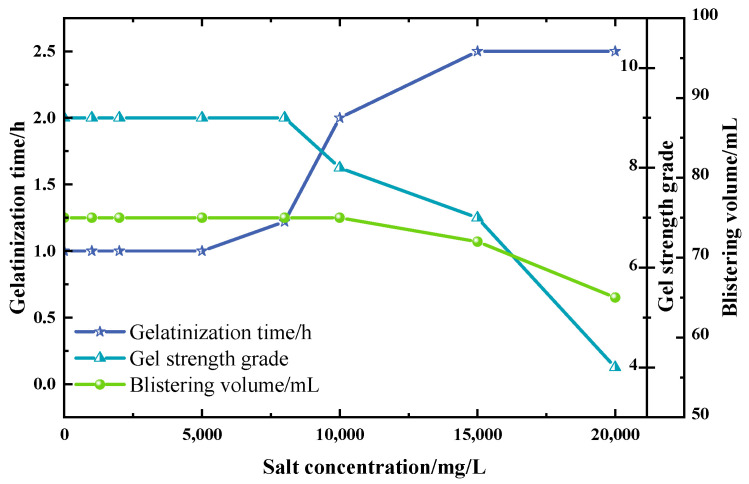
Effect of salt concentration on the properties of foam gels.

In this study, a 50 mL polyvinyl alcohol (PVA) foam gel base solution was prepared, comprising 4 wt% polyvinyl alcohol (PVA), 3 wt% titanium crosslinking agent (TiJ), and 0.7 wt% gas generator V-50. During the experiment, the salinity was adjusted from 1000 mg/L to 20,000 mg/L, increasing at specific intervals, to investigate the effect of salinity on the performance of the foam gel. The experimental results are presented in Figure 11.

As illustrated in Figure 11, the gelation time of the polyvinyl alcohol foam gel progressively increases with the rise in salinity. At a salinity of 10,000 mg/L, the foaming volume is reduced, and the gel grade drops from grade I to grade H, yet it continues to exhibit a high level of strength. With the increase in salinity, the concentration of Na^+^ increases, leading to competition between metal ions and the active groups on the crosslinking agent, thereby extending the gelation time. Moreover, at high salinity levels, metal ions form a substantial salt layer on the polymer molecular chain, which disrupts some of the spatial structure of the gel, resulting in a modest reduction in the gel grade. Considering both the gelation time and gel strength, the observation results indicate that the polyvinyl alcohol foam gel exhibits good salt tolerance at a salinity of 15,000 mg/L.

#### 2.3.2. Effect of Gelation Temperature

**Figure 12:**
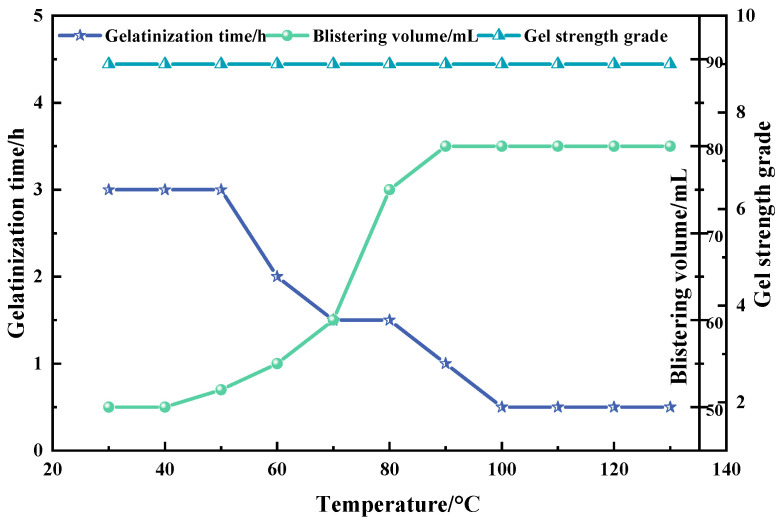
Effect of temperature on the properties of foam gels.

In this study, a 50 mL polyvinyl alcohol foam gel base solution was prepared, comprising 4 wt% polyvinyl alcohol (PVA), 3 wt% titanium crosslinking agent (TiJ), and 0.7 wt% gas generator V-50. During the experiment, the temperature was set within the range of 30 °C to 130 °C, with intervals of 10 °C, to investigate the effect of temperature on the performance of the foam gel. The experimental results are presented in Figure 12.

As illustrated in Figure 12, the gelation time of the polyvinyl alcohol foam gel progressively decreases as the temperature rises; the foaming volume initially increases and then stabilizes with the increase in temperature; the gel grade remains constant throughout the process of temperature change. As the temperature rises, it enhances the hydrogen bonds and certain chemical bonds of lower bond energy within the gel crosslinked structure, resulting in a reduced gelation time. At lower temperatures, the foaming agent azobisisobutyronitrile hydrochloride (V-50) absorbs less thermal energy during decomposition, resulting in the majority of the foaming agent remaining undecomposed. As the temperature increases, more foaming agent decomposes to produce gas, increasing the foaming volume. At 90 °C, the foaming agent completely decomposes, and the foaming volume reaches its maximum. Further increases in temperature do not cause changes in the foaming volume. Even at 130 °C, the foam gel does not decompose, maintaining a high strength, demonstrating good temperature resistance.

#### 2.3.3. Viscoelastic Analysis

**Figure 13:**
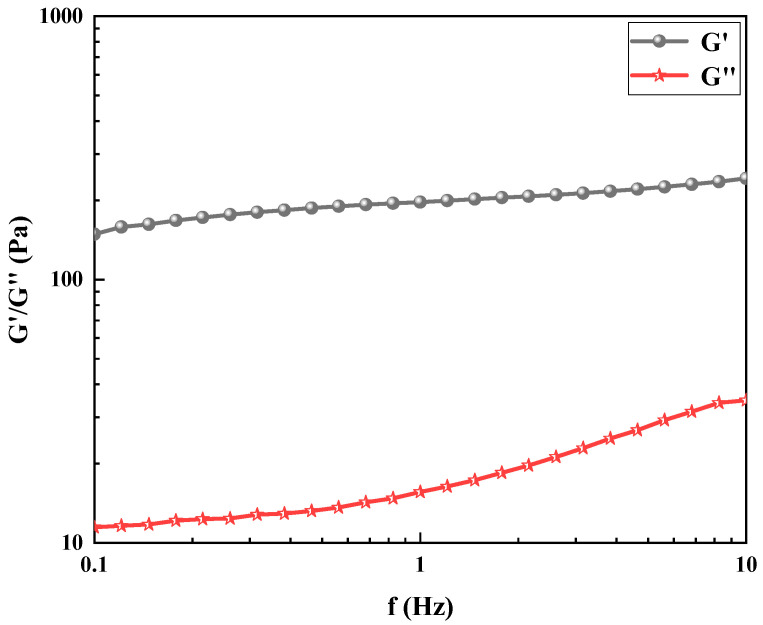
Viscoelastic analysis of gels.

This study prepared a 50 mL polyvinyl alcohol foam gel base solution, consisting of 4 wt% polyvinyl alcohol (PVA), 3 wt% titanium crosslinking agent (TiJ), and 0.7 wt% gas generator V-50. The storage modulus (G’) and loss modulus (G”) of the foam gel were measured at different shear frequencies (f) using a rheometer. The relevant experimental results are presented in Figure 13.

Rheological properties are important indicators for characterizing the viscoelastic features and mechanical strength of hydrogels. The storage modulus (G’) indicates the elastic portion of the gel. When the storage modulus is greater than the loss modulus, it indicates that the material behaves as an elastic solid. As shown in Figure 13, the storage modulus and loss modulus of the polyvinyl alcohol foam gel increase gradually with the increase in shear frequency. The storage modulus is always greater than the loss modulus. This experimental phenomenon demonstrates that crosslinking has occurred between polyvinyl alcohol and the organic titanium crosslinker, resulting in the formation of a gel.

#### 2.3.4. Long-Term Stability

**Figure 14:**
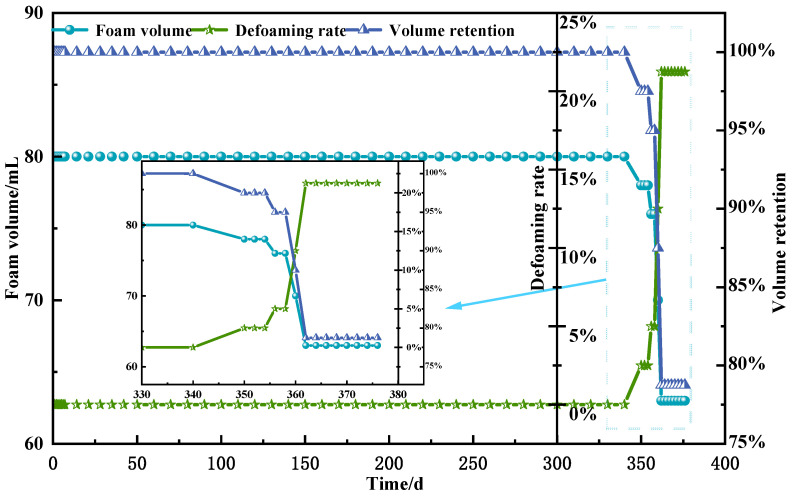
Relationship between volume retention rate and defoaming rate with time.

This study prepared a 50 mL polyvinyl alcohol foam gel base solution, consisting of 4 wt% polyvinyl alcohol (PVA), 3 wt% titanium crosslinking agent (TiJ), and 0.7 wt% gas generator V-50. The samples were placed in a constant temperature oven set at 90 °C, and the changes in the volume and gel strength of the foam gel over time were observed. This experiment aims to assess the long-term stability of the foam gel at high temperatures by analyzing the volume retention rate and defoaming rate of the foam gel. The relevant experimental results are presented in Figure 14.

The foam gel system is characterized as an unstable fluid that evolves over time, with its total volume determined by the foam gel base solution and the amount of gas produced by the foaming agent. Over time, the rupture of the foam structure leads to the escape of gas, thereby causing a reduction in the total volume of the foam gel. As illustrated in Figure 14, the polyvinyl alcohol foam gel prepared in this study maintained a constant volume for the initial 340 days. In the subsequent 22 days, due to the progressive breakdown of the foam structure and the release of gas, the volume of the gel began to decline. At 362 days, the volume retention rate was 78.75%. This result indicates that the prepared polyvinyl alcohol foam gel exhibits excellent long-term stability. Compared to traditional foam gels, it exhibits better long-term stability [24,25].

#### 2.3.5. Plugging Performance

**Figure 15:**
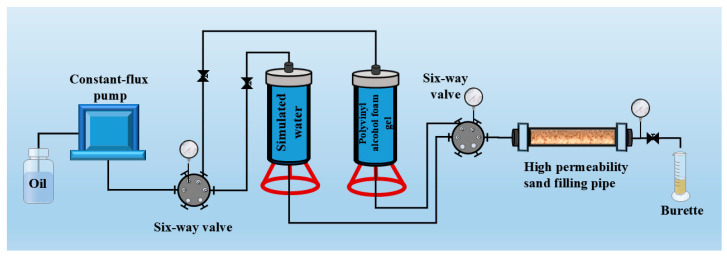
Schematic diagram of the displacement experimental setup.

To evaluate the plugging performance of the polyvinyl alcohol foam gel, a displacement device (as shown in Figure 15) was used. The initial permeability of the sand-packed tube was determined through a primary water drive. Subsequently, the polyvinyl alcohol foam gel base solution was injected into the sand-packed tube at a rate of 2 mL/min. The solution was maintained at 90 °C for 24 h to promote gas generation, followed by a secondary water drive experiment. The plugging performance data of the polyvinyl alcohol foam gel are presented in Table 2. The original permeability of the sand-packed tube was 851 mD. After the gel was injected, the permeability during the secondary water drive was significantly reduced to 31 mD, with a plugging efficiency of 96.36%, which is higher than that of other high-strength gel foams [14,15]. These results indicate that the polyvinyl alcohol foam gel base solution has good injectability and can effectively plug the sand-packed tube model.

## 3. Conclusions

(1) By adjusting the polyvinyl alcohol (PVA) concentration from 2 wt% to 5 wt%, the crosslinking agent concentration from 2.5 wt% to 5 wt%, and the gas generator concentration from 0.3 wt% to 0.9 wt%, the foam gel can be tailored to achieve a gel strength ranging from grade G to grade I, a gelation time from 0.2 to 6 h, and a gelation volume from 50 to 80 mL.
(2) Within the temperature range of 30 to 130 °C, the gel strength of the foam gel remains constant, demonstrating good temperature resistance. At 90 °C, the foam gel can stably exist for up to 340 days with minimal strength variation.
(3) The results of the plugging experiment indicate that the prepared foam gel system possesses excellent injectability and is capable of effectively sealing the sand-packed tube model, achieving a plugging efficiency of up to 96.36%.

## 4. Materials and Methods

### 4.1. Materials

Polyvinyl alcohol (PVA1788, 1797, 1799 types), AR, Shanghai Macklin Biochemical Technology Co., Ltd., Shanghai, China; 2,2′-azobis(2-methylpropionamidine) dihydrochloride (V-50), AR, Shanghai Macklin Biochemical Technology Co., Ltd., Shanghai, China; Sodium chloride, AR, National Pharmaceutical Group Chemical Reagent Co., Ltd., Shanghai, China; Organic titanium crosslinking agent, self-made in the laboratory [26,27,28,29].

### 4.2. Preparation of Polyvinyl Alcohol Foam Gel

Prepare 50 mL of a polyvinyl alcohol (PVA) solution with a specific concentration using distilled water. Add a predetermined amount of crosslinking agent and gas-generating agent to the solution and mix thoroughly to ensure complete dissolution. Transfer the prepared solution to a 100 mL graduated cylinder with a stopper and place it in a water bath at various temperatures to observe the gel strength, gelation time, and gel volume.

### 4.3. Evaluation Methods for Polyvinyl Alcohol Foam Gels

(1) Gel Strength. Gel strength is defined as the ability of a gel to resist deformation and destruction under the action of force. The gel strength of the foam gel is evaluated using the gel code method [30].
(2) Gelation Time. The time required for the gel strength to reach grade F—highly deformable non-flowing gel.
(3) Gel Volume. The volume of the gel after it has fully expanded and no longer increases, representing the maximum volume achieved post-gelation.
(4) Volume Retention Rate. The ratio of the remaining volume over time to the initial volume is defined as the volume retention rate.
(5) Defoaming Rate. The ratio of the volume reduction of foam gel over time to the initial volume is defined as the defoaming rate.

### 4.4. Characterization Methods for Polyvinyl Alcohol Foam Gels

(1) FTIR. The samples were first freeze-dried and then analyzed using the Thermo Fisher Scientific Nicolet 6700 FTIR spectrometer (Waltham, MA, America) with the KBr pellet method.
(2) SEM. The gel samples were freeze-dried. The microstructure of the gel was analyzed using a MIRA3 field emission scanning electron microscope of TESCAN Trading Co., Ltd. (Shanghai, China) after gold-coating.
(3) Microstructure of the Gel. A small quantity of foam gel was placed onto a glass slide. The slide was fixed with a spring clip, and the focus was adjusted using a 4× objective lens for observation. Observations through the eyepiece were made with the left eye. If a blurry image appeared, the fine adjustment knob was gently rotated counterclockwise by 0.5–1 turn to achieve a clear image. The experiment used an FY800 optical microscope from Jinan Fangyuan Testing Instruments Co., Ltd. (Jinan, China) for observation.
(4) TG. The foam gel samples were subjected to freeze-drying, followed by grinding, weighing, and placing into a crucible. The thermal gravimetric (TG) curve of the sample was measured using the SETARAM (Lyon, France) LABSYS evo TGA. The measurement was conducted in an N_2_ atmosphere, with a temperature range of 30–600 °C and a heating rate of 5 °C/min.
(5) Viscoelastic Analysis. The rheological properties of the foam gel samples were tested using the rotational rheometer (model MCR92) from Anton Paar (Graz, Austria). An appropriate amount of the sample was placed on the sample stage. The test rotor used was a parallel plate with a diameter of 25 mm, and the gap was set to 1 mm. Frequency sweep mode was employed, with a frequency range of 0.1–10 Hz. A constant strain of 1% was applied to measure the variation trends of the storage modulus and loss modulus.

## Figures and Tables

**Table 1:** Viscosity of different types of polyvinyl alcohol 5 wt% solutions.

PVA Types	Viscosity/mPa·s
PVA 1788	61.40
PVA 1797	48.51
PVA 1799	84.12

**Table 2:** Experimental results of foam gel system plugging.

Test Items	Results
Viscosity of foam gel base/mPa·s	182.47
Pressure difference of primary water flooding ∆P1/MPa	0.21
Permeability of primary water flooding K1/mD	851
Differential pressure of secondary water flooding ∆P2/MPa	5.78
Permeability of secondary water flooding K2/mD	31
Blocking rate %	96.36

## Data Availability

The original contributions presented in this study are included in the article. Further inquiries can be directed to the corresponding author.
